# Low-frequency noise of MoTe_2_ transistor: effects on ambipolar carrier transport and CYTOP doping

**DOI:** 10.1186/s11671-024-04068-8

**Published:** 2024-11-19

**Authors:** Wonjun Shin, Dong Hyun Lee, Raksan Ko, Ryun-Han Koo, Hocheon Yoo, Sung-Tae Lee

**Affiliations:** 1 Inter-University Semiconductor Research Center, Department of Electrical and Computer Engineering, Seoul National University, Seoul, 08826 Republic of Korea; 2 Department of Semiconductor Convergence Engineering, Sungkyunkwan University, Suwon, Gyeonggi-do 16419 Republic of Korea; 3 Department of Semiconductor Engineering, Gachon University, Seongnam, 13120 Republic of Korea; 4 School of Electronic and Electrical Engineering, Hongik University, Seoul, 04066 Republic of Korea; 5 Department of Electronic Engineering, Gachon University, Seongnam, 13120 Republic of Korea

**Keywords:** Ambipolar transistors, CYTOP, MoTe_2_, Low-frequency noise

## Abstract

Low-frequency noise (LFN) characteristics of semiconductor devices pose a significant importance for understanding their working principle, particularly concerning material imperfections. Accordingly, substantial research endeavors have focused on characterizing the LFN of devices. However, the LFN characteristics of the ambipolar transistors have been rarely demonstrated. Herein, we investigate the effects of ambipolar carrier transport and CYTOP-induced *p*-type doping on low-frequency noise characteristics of MoTe_2_ transistors. The source of the 1/*f* noise differs between the *n*-type (electron transport) and *p*-type (hole transport) modes. Notably, the influence of contact resistance is more pronounced in the *n*-type mode. CYTOP doping suppresses the *n*-type mode by introducing hole doping effects. Furthermore, CYTOP doping mitigates the impact of contact resistance on excess noise.

## Introduction

As Moore’s law that has been based on continuous scaling on the silicon field-effect-transistors reaches its limit, there has been a notable surge in research focused on 2D materials as potential foundational elements for future electronics [1–3]. Among these materials, graphene has emerged as a focal point of semiconductor investigation due to its manifold advantages over silicon, including heightened electron mobility, superior thermal conductivity, inherent flexibility, and the potential for diminutive design afforded by its monolayer configuration. Nonetheless, the intrinsic lack of a discernible band gap in graphene poses a substantial impediment to its utility in logic circuits and diverse applications. Accordingly, other 2D materials and their derivatives, such as transition-metal dichalcogenides (TMDs), exhibit remarkable electrical performance and hold significant promise for the development of post-silicon devices [4–6]. Unlike graphene, TMDs such as MoS_2_, MoSe_2_, and MoTe_2_ possess a finite band gap, rendering them suitable for applications. MoTe_2_, in particular, stands out with its impressive performance, featuring a band gap ranging from 0.81 eV (indirect) in bulk form to 1.13 eV (direct) in monolayer [7–9]. This transition from indirect to direct band gap is significant as it enhances the material's optoelectronic properties, making it suitable for applications such as photodetectors and light-emitting devices. Moreover, in MoTe_2_, the band gap energy approximately corresponds to the combined Schottky barrier energies for both electrons and holes. This characteristic, combined with the small band gap of MoTe_2_, facilitates carrier injection for both electrons and holes, resulting in ambipolar characteristics.

While the ambipolarity of MoTe_2_ transistors offers potential for various applications [10, 11], it is crucial to have control over this property to harness its benefits effectively. One approach to modulating the ambipolar effects is through doping the MoTe_2_ [12–14]. Doping introduces impurities into the material, altering its electrical properties. However, the research for an effective doping technique for TMDs, including MoTe_2_, remains largely unexplored. Traditional methods, such as ion implantation, are incompatible with TMDs due to their tendency to cause significant damage to the crystal structure, thereby compromising the material stability. Alternatively, one can consider substituting the transition metals or chalcogen atoms in TMDs with different elements [12]. This substitution approach offers a promising route to tailor the electronic structure of MoTe_2_, albeit it requires precise control over the substitution process to achieve desired properties reliably. Gas doping presents another avenue to explore, where exposure to gases like NO_2_ or K can modify the electrical properties of TMDs [13]. However, these approaches involve complex procedures and suffer from poor reliability and reproducibility, posing challenges for scalable device fabrication and commercialization. Thus, there is a pressing need for innovative doping techniques that can effectively modulate the ambipolar behavior of MoTe_2_ transistors while ensuring device performance, reliability, and scalability. Recently, a simple *p*-doping method utilizing spin-coating of CYTOP onto TMDs has been introduced [14]. CYTOP is a fluoropolymer that dissolves in exclusive fluorine-based solvents and this technique creates composite material comprising TMDs and a fluoropolymer with precise control over the electrical polarity of TMDs. However, a comprehensive examination of the electrical properties of the doped devices is still lacking. In this context, low-frequency noise (LFN) analysis with high sensitivity to changes in electrical properties [15–17] is required for accurate analysis of conduction mechanism and the impact of doping. LFN characterization involves analyzing the fluctuations in electrical signals at frequencies lower than conventional measurements. It provides valuable insights into carrier mobility, density-of-states, interface traps, and material imperfection crucial for understanding device behavior. By subjecting the doped MoTe_2_ transistors to LFN analysis, researchers can assess the effectiveness of the doping process, quantify changes in electrical properties, and optimize device performance. Additionally, LFN analysis enables the identification of potential sources of noise and instability in the devices, aiding in the development of strategies to mitigate these issues and enhance device reliability. Thus, integrating LFN characterization into the study of doped MoTe_2_ transistors promises to advance our understanding of their electrical behavior and accelerate the development of next-generation electronic devices. There have been several studies on the LFN characteristics of MoTe_2_ FETs, including the effects of ambient conditions (air or vacuum) [15], double gate structure [16], and CYTOP passivation effects [17]. However, the effects of doping effects on ambipolar behavior with respect to LFN characteristics have not been systematically demonstrated.

In this study, we investigate the LFN characteristics of the MoTe_2_ in conjunction with material analysis, including Raman spectroscopy and surface energy analysis. Mechanically exfoliated MoTe_2_s are transferred to fabricate the transistor and the LFN of the MoTe_2_ transistors is characterized by measuring the power spectral density (PSD) of the devices at different operating regions. The measurement results are fitted into carrier number fluctuation (CNF) and Hooge’s empirical models that have been widely used for explaining the possible noise sources within semiconductor transistors. The results illustrate distinct conduction and noise generation mechanisms depending on the carrier transport type. Furthermore, we demonstrate the impact of CYTOP doping on the LFN characteristics of the MoTe_2_ transistors. It is revealed that the ambipolar characteristics of the device are improved, and a corresponding reduction in 1/*f* noise is achieved.

## Material and methods

**Fig. 1:**
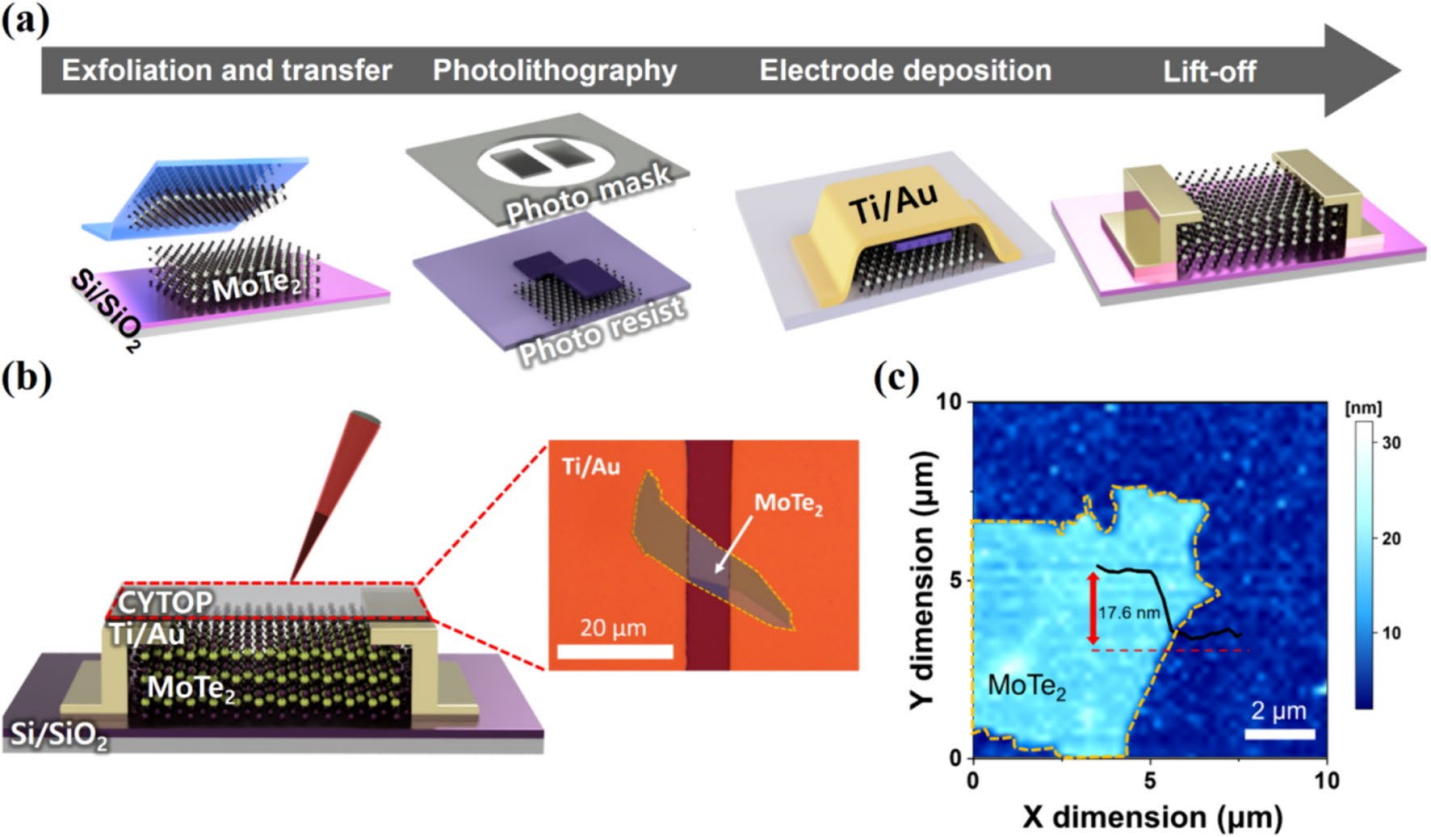
**a** Fabrication process of MoTe_2_ transistor fabricated through mechanical exfoliation. **b** 3D schematic of CYTOP doped MoTe_2_ transistor and top-view optical microscopy image. **c** Atomic force microscope analysis of multilayer MoTe_2_ flake

Figure 1a shows the schematic diagram of the fabrication process for the MoTe_2_ transistors. Multilayer MoTe_2_ flakes were obtained through mechanical exfoliation from bulk MoTe_2_ crystals (HQ Graphene, Netherlands) and subsequently transferred a Si/SiO_2_ substrate with a 300 nm thick SiO_2_ layer (Namkang Hi-Tech Inc., South Korea). Subsequently, source and drain electrodes were deposited on the transferred substrate using a conventional photolithography method (EVG 620, EV Group, Austria). A 20 nm of Ti and 80 nm of Au layers were deposited via an electron beam evaporator (SHE-8T-500, Samhan Vacuum Development, South Korea), followed by a lift-off process. The fabricated MoTe_2_ transistors underwent a spin-coating process with a CYTOP solution (solvent-to-polymer ratio = 1: 10) at 3000 rpm for 60 s. Sequential annealing on a hot plate was then performed at 120 and 150 °C for 30 min each. Figure 1b presents a 3D schematic of the MoTe_2_ transistor doped with CYTOP. Additionally, the thickness and surface morphology of the multilayer MoTe_2_ flakes were characterized using atomic force microscopy (Fig. 1c). The results confirm the thickness of the multilayer MoTe_2_ flakes to be 17.6 nm.

## Results and discussion

To characterize the LFN of the MoTe_2_ transistors, the PSD is measured using a semiconductor device parameter analyzer B1500, a current preamplifier SR570, and a signal analyzer 35670A. The gate and drain electrodes were connected to the B1500 for bias applications. The output current from the device was then connected to the SR570 for amplification. Subsequently, the amplified signal was fed into the 35670A, which performed a Fast Fourier Transform to convert the time-domain signal and fluctuation into the frequency domain. This facilitated the calculation of the PSD, a crucial metric for analyzing the LFN characteristics. To ensure the reliability and reproducibility of the collected data, five independent devices were utilized in the study. For each device, the PSD measurement was repeated three times, and the results were averaged. This approach helps to minimize experimental variability and provides a robust dataset for analysis.

**Fig. 2:**
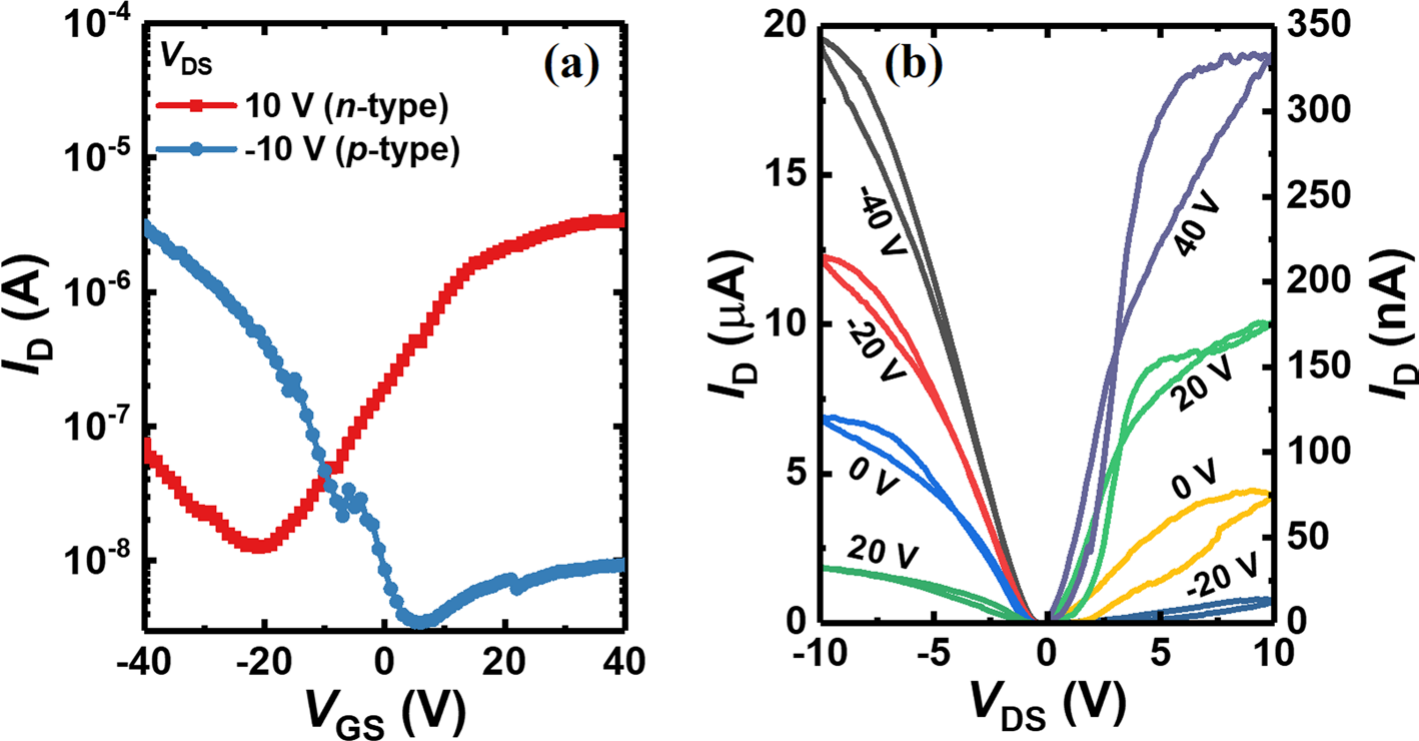
**a** Transfer and **b** output characteristics of the pristine MoTe_2_ transistors in *n*- and *p*-type modes, respectively

The electrical characteristics of the fabricated MoTe_2_ are demonstrated by measuring the transfer and output characteristics. Figure 2a shows the transfer characteristics (*I*_D_-*V*_GS_) of the pristine MoTe_2_ transistors without CYTOP doping. The measurements are conducted by varying the gate-source bias (*V*_GS_) from − 40 to 40 V while keeping the drain-source bias (*V*_DS_) fixed at 10 V (− 10 V) to observe the *n* (*p*)-type operation. The ambipolar characteristics are clearly demonstrated in the MoTe_2_ transistors because the band gap energy approximately corresponds to the combined Schottky barrier energies for both electrons and holes. Figure 2b shows the output characteristics (*I*_D_-*V*_DS_) of the MoTe_2_ transistors. The field-effect mobility (${\mu }_{eff})$ of MoTe_2_ transistors is expressed as [18, 19]

1 $$\mu_{eff} = \frac{{\partial I_{D} }}{{\partial V_{G} }}\frac{L}{{WC_{ox} V_{D} }} = \frac{{g_{m} L}}{{WC_{ox} V_{D} }}$$

where *L* and W are the channel length and width, respectively. *C*_ox_ is the gate oxide capacitance, and *g*_m_ is the transconductance of the MoTe_2_ transistor. The calculated *p*- and *n*-type mobility of the ambipolar MoTe_2_ transistor is 1.7 cm^2^/V·s and 1.1 cm^2^/V·s, respectively, which shows a balanced coexist of electron and hole carrier transport.

**Fig. 3:**
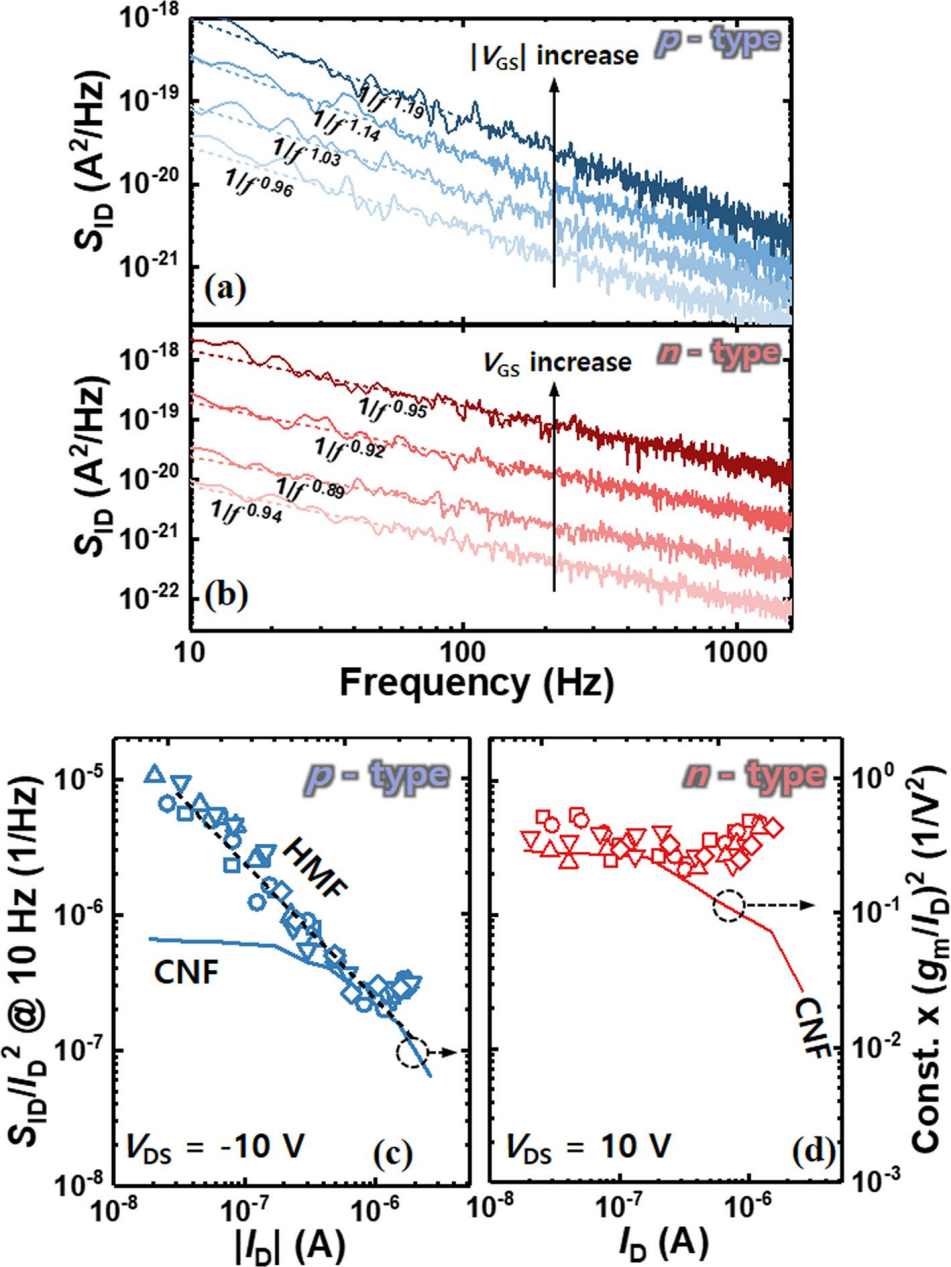
*S*_ID_/*I*_D_^2^ versus *f* for the MoTe_2_ transistors in **a** *p*- and **b** *n*-type operations, respectively. *S*_ID_/*I*_D_^2^ and (*g*_m_/*I*_D_)^2^ versus *I*_D_ for the MoTe_2_ transistors in **c** *p*- and **d** *n*-type operations, respectively

Now, we investigate the LFN characteristics of the MoTe_2_ transistors. In order to examine the origin of the LFN within semiconductor transistors, how the PSD changes depending on the bias changes should be demonstrated. The most important is to demonstrate the gate bias dependence of the device. Figure 3a and b show the drain current (*I*_D_) power spectral density (PSD) (*S*_ID_) versus frequency (*f*) for the MoTe_2_ transistors in *p*- and *n*-type operations, respectively. The PSDs are obtained by varying the *V*_GS_, while fixing the *V*_DS_ to − 10 or 10 V. In both *p*- and *n*-type operations, the MoTe_2_ transistors exhibit 1/*f* noise behavior. To demonstrate the underlying source of 1/*f* noise in each operation mode, we examine the correlation between the *S*_ID_/*I*_D_^2^ and the square of the transconductance-to-drain current ratio ((*g*_m_/*I*_D_)^2^) as a function of *I*_D_. Figure 3c and d show *S*_ID_/*I*_D_^2^ and (*g*_m_/*I*_D_)^2^ versus *I*_D_ for the MoTe_2_ transistors in *p*- and *n*-type operations, respectively. Note that the measurements are repeated five times for repeatability. In the *p*-type mode MoTe_2_, the *S*_ID_/*I*_D_^2^ and (*g*_m_/*I*_D_)^2^ exhibit different behavior for *I*_D_. Instead, the *S*_ID_/*I*_D_^2^ demonstrates a decreasing trend with a slope of − 1 in the low *I*_D_ region, followed by an increase in the high *I*_D_ region. The decreasing trend in the low *I*_D_ region indicates that the 1/*f* noise originates from the carrier mobility fluctuation within the bulk of the MoTe_2_ channel, which can be explained by Hooge’s empirical model [19, 20]. This is expressed as

2 $$\frac{{S}_{ID}}{{{I}_{D}}^{2}}=\frac{{\alpha }_{H}{\mu }_{eff}2kT}{f{L}^{2}{I}_{D}}$$

where α_H_ is Hooge’s parameter, *k* is the Boltzmann constant, *T* is the temperature. The subsequent increase in the high *I*_D_ region indicates that contact resistance plays a significant role in determining the conduction mechanism [20]. Accordingly, the noise is mainly generated by the Schottky barrier height fluctuation at the contact area.

**Fig. 4:**
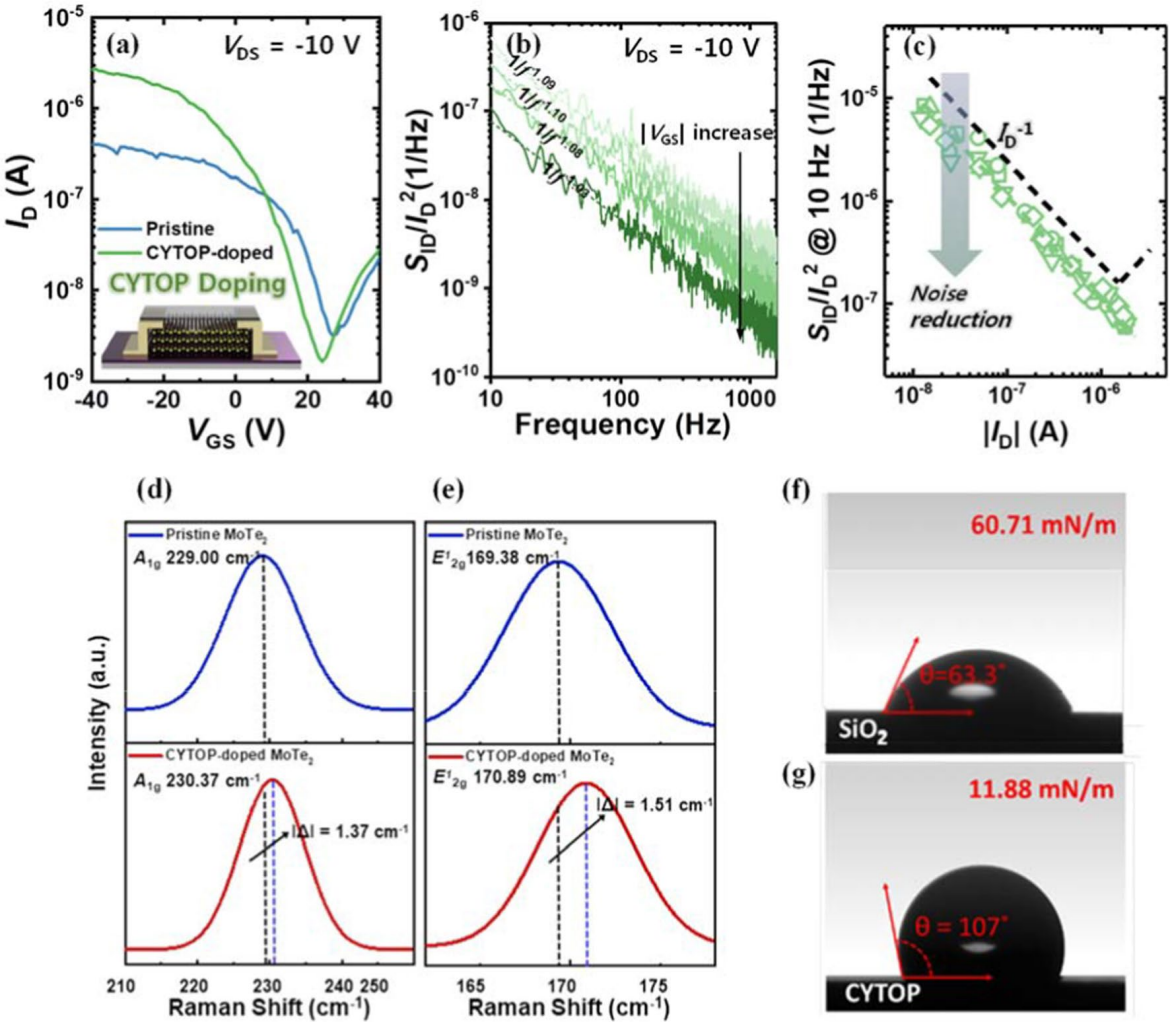
**a** Transfer characteristics of the MoTe_2_ transistors with and without CYTOP doping. **b** *S*_ID_/*I*_D_^2^ versus *f* for the CYTOP doped MoTe_2_ transistor. **c** *S*_ID_/*I*_D_^2^ sampled at 10 Hz versus *I*_D_ of the device. Raman peak shift data for **d** A_1g_ and **e** E^1^_2g_ modes. Surface energy and contact angle images of deionized water **f** before and **g** after CYTOP doping

Contrarily, in the *n*-type MoTe_2_ transistor, the *S*_ID_/*I*_D_^2^ demonstrates a similar behavior to (*g*_m_/*I*_D_)^2^ in the low *I*_D_ region. This behavior can be elucidated by the carrier number fluctuation (CNF) model [21–23]. According to the CNF model, the 1/*f* noise arises from the random trapping and detrapping process of channel carriers to and from gate oxide. The CNF model is expressed as [21]

3 $$\frac{{S}_{ID}}{{I}_{D}^{2}}={(\frac{{g}_{m}}{{I}_{D}})}^{2}\frac{{q}^{2}{k}_{B}T{N}_{T}\lambda }{WL{{C}_{ox}}^{2}f}$$

where *N*_T_ is volume trap density, $\lambda$ is tunneling attenuation coefficient,. The MoTe_2_ transistor exhibits an intriguing observation where the 1/*f* noise exhibits distinct noise sources depending on the carrier type. This discrepancy can be attributed to the bulk conduction characteristics of the hole carriers, while the electrons are predominantly located closer to the gate oxide channel interface. Note that the value of N_T_ from CNF model of the n-type MoTe_2_ transistor is 8.7 $\times$ 10^20^ cm^−3^ eV^−1^. Additionally, there is a notable disparity in the behavior of *S*_ID_/*I*_D_^2^ between the *n*- and *p*-type MoTe_2_ transistors. The increase in *S*_ID_/*I*_D_^2^ is observed at a much lower *I*_D_ in the *n*-type MoTe_2_ transistor compared to the *p*-type transistor. This finding indicates that the contact resistance and associated noise are more significant in the *n*-type mode. A quantitative analysis of the contact resistance noise can be evaluated by adding the contact noise term in Eq. (3). This term is given as ${(\frac{{I}_{\text{D}}}{{V}_{\text{DS}}})}^{2}{S}_{R\text{sd}}$, where *S*_Rsd_ is the spectral density of source-drain series resistance In the *n*-type device, the *S*_Rsd_ is 21.3 Ω^2^/Hz. Now, we investigate the impact of CYTOP doping on the LFN characteristics. Significant improvements are observed in both current and hysteresis after the CYTOP doping (Fig. 4a). Note that the *n*-type behavior is absent within the *V*_GS_ sweep range of − 40 to 40 V at *V*_DS_ = 10 V (not shown). This suggests that the CYTOP doping effectively suppresses the *n*-type behavior in the device. **[R2C6]** Also, the CYTOP doping on pristine MoTe_2_ transistor induced hole doping effects. The hole field-effect mobility of the pristine and CYTOP-doped MoTe_2_ transistors is 0.06 cm^2^/V·s and 0.49 cm^2^/V·s, respectively, which increases approximately 8 times after CYTOP is introduced. Figure 4b shows the *S*_ID_/*I*_D_^2^ versus *f* for the CYTOP-doped MoTe_2_ transistor. Figure 4c shows the *S*_ID_/*I*_D_^2^ at 10 Hz versus *I*_D_ of the transistors with CYTOP doping (green open symbols) and without CYTOP doping (black dashed line). The LFN characteristics of the CYTOP-doped MoTe_2_ transistor are explained by Hooge’s empirical model. Moreover, the magnitude of 1/*f* noise is decreased across all *I*_D_ regions and the contact-induced excess noise is suppressed by CYTOP doping. This can be attributed to the reduction in carrier mobility scattering and contact-associated charge fluctuations by CYTOP doping. Note that the where α_H_ is decreased from 2.5 $\times$ 10^–1^ to 7.4 $\times$ 10^–2^ by CYTOP doping, demonstrating the improved noise characteristics.

To further explore the impact of CYTOP doping, we conduct a material analysis of MoTe_2_. Raman spectroscopy, a widely employed method for confirming doping effects in 2D materials [24], is utilized to investigate the *p*-doping effect of CYTOP on the devices (Fig. 4d and e). Following CYTOP doping, the A_1g_ and E^1^_2g_ peaks exhibit blue shifts of 1.37 cm^−1^ and 1.51 cm^−1^, respectively, demonstrating the *p*-doping effects on MoTe_2_ [25]. The increase in hole carrier concentration by the doping aligns with the LFN measurement results that exhibit a decrease in 1/*f* noise (Fig. 4b). Subsequently, we delve into the influence of CYTOP on contact angle and surface energy [26]. Figure 4f and g show the contact angles and surface energy of deionized water on pristine SiO_2_ (θ = 63.3°, 60.71 mN/m) and CYTOP-doped SiO_2_ (θ = 107°, 11.88 mN/m). This result indicates a conversion of the SiO_2_ surface from hydrophilic to hydrophobic through CYTOP doping. Pristine SiO_2_, possessing a high surface energy, harbors numerous active sites that can interact with molecules and ions in the atmosphere. It seems that CYTOP doping reduces surface energy, thereby alleviating the 1/*f* noise associated with the mobility scattering of bulk-conducting hole carriers induced by interface trap sites. These results are in alignment with the LFN characterization of the MoTe_2_ transistors. We believe that the results in this work contribute to the development of LFN characterization of transistors with novel materials and device systems, including 2D semiconductors [27–29], organic semiconductors [30–32], metal oxide [33–35], ferroelectric [36–38], gas sensors [39–41], and synaptic devices [42–45].

## Conclusions

In this study, we demonstrated the LFN characteristics of MoTe_2_ transistors, focusing on ambipolar carrier transport and the CYTOP doping effect. The primary contributor to noise in the *n*-type mode is the CNF, whereas, in the *p*-type mode, it stems from carrier mobility fluctuation. Furthermore, our findings indicate that CYTOP doping effectively inhibits electron transport and reduces the magnitude of 1/*f* noise by hole carrier doping and surface energy reduction. The results in this study not only provide a valuable insight of LFN characteristics of ambipolar MoTe_2_ transistors but also demonstrate the doping effects on carrier transport mechanism of the device.

## Data Availability

The datasets used and/or analyzed during the current study are available from the corresponding author on reasonable request.
